# The unusual finding of peripheral lymphadenopathy among confirmed Lassa fever patients in Nigeria

**DOI:** 10.2144/fsoa-2022-0086

**Published:** 2023-05-01

**Authors:** Sampson O Owhin, Chukuyem Abejegah, Lanre O Olatunde, Nelson A Adedosu, Olufemi O Ayodeji, Timothy R Folorunso, Joachim Azegbeobor, Peter E Akhideno, George O Akpede, Joseph A Ayeyemi, Oyebanji Z Olowosusi, Cyril Erameh, Liasu A Ahmed

**Affiliations:** 1 Federal Medical Center, Owo, 341101, Adekunle Ajasin road, Owo, Ondo State, Nigeria; 2 Irrua Specialist Teaching Hospital, Irrua, KM 87, Benin Auchi Expressway, 310115, Edo State, Nigeria

**Keywords:** clinical features, Federal Medical Center Owo (FMCO), Lassa fever, lymphadenopathy

## Abstract

Lassa fever is a viral haemorrhagic fever belonging to the arenaviridae family that is well known to be endemic to West Africa. The clinical presentation of the disease ranges from asymptomatic to fulminant illness. Lymphadenopathy a clinical manifestation of inflammation, infection, or malignancy has not been widely reported in Lassa fever disease. We report two cases of Lassa fever disease presenting with lymphadenopathy.

## Plain language summary

Enlargement of lymph nodes, is a common symptom of many infections, however it is not commonly mentioned in patients with Lassa fever, a viral hemorrhagic fever that is endemic in West Africa. However, recent research suggests that lymphadenopathy may be underreported in Lassa fever patients. This new finding could have important implications for the diagnosis and treatment of the disease, as well as for our understanding of how it spreads.

Lassa fever virus disease (LFVD) is an acute viral hemorrhagic fever caused by the Lassa fever virus [1–3], which is a single-stranded RNA virus belonging to the genus *Arenaviridae*. The reservoir host is the multimammate rat, *Mastomys natalensis* and transmission is through contact with blood, urine, or the excreta of infected rats [4, 5] but could also be acquired through contact with blood and other body fluids of infected persons [5]. Transmission could occur in families and health facilities with poor knowledge of the disease and practice of preventive measures [6].

Lassa fever was first identified in Lassa town, Borno State, northeastern Nigeria in 1969 but is endemic in many West African countries including Nigeria, Sierra Leone, Liberia, Guinea, Benin and Mali [2]. It is responsible for yearly epidemics in West Africa with estimated 300,000–500,000 cases and 5000 deaths per annum [2]. Sporadic cases are also seen all year round in endemic areas [4]. Both epidemic and sporadic outbreaks have devastating public health and socio-economic implications [4] and mortality could be as high as 65% among severely ill hospitalized patients during outbreaks [4, 7–9].

## Clinical features of Lassa fever

Those who contract the disease may not manifest with any significant symptom while others may present with severe systemic manifestations [9].

The clinical presentations of LFVD are not pathognomonic and as such it poses a diagnostic conundrum with other tropical diseases with similar manifestations such as malaria and Ebola. However, diagnosis of LFVD should be considered in patients presenting with fever (≥38°C)who are not responding adequately to antimalarial and antibiotic drugs. As reported in a study, early features such as: fever, sore throat, retrosternal pain, headache and presence of protein in urine could play a major role at prompt diagnosis. While late features such as: mucosal bleeding (17%), sensorineural hearing deficit (4%), pleural effusion (3%), pericardial effusion (2%), oedema of the face and neck are often associated with poor outcomes [10–12].

## Lymphadenopathy

One of the components of the immune system is the lymphatic system, a complex component involved in filtering substances in the body. Lymphocytes are white blood cells involved in searching for specific proteins and travel through lymph nodes, which are widely placed throughout the body. Lymphadenopathy is a term that refers to the enlargement of lymph nodes. Lymph nodes are small glands that are responsible for filtering fluid from the lymphatic system. They are divided into sections known as follicles, which are subdivided into B zones and T zones, which represent the base location of lymphocytic maturation [13].

Inflammation, infection, or malignancy, may result in the unusual proliferation of lymph nodes and thus, clinicians must perform a detailed history and physical examination to screen for lymphadenopathy, as this may help in increase diagnostic accuracy. A detailed examination of specific anatomic regions of the body, including the neck, supraclavicular, axillary, and inguinal regions for lymphadenopathy should be considered. It should be noted that the size of a normal lymph node in the adult population should be less than 1 cm; however, there are exceptions to this rule [13].

Lymphadenopathy involving two or more noncontiguous sites, also known as generalized lymphadenopathy occurs in only 25% of cases as opposed to localized lymphadenopathy, which is responsible for 75% of cases [14].

The Lassa virus is known to infect immune cells such as dendritic cells, macrophages, and T-cells. When these cells become infected, they release cytokines and chemokines that attract other immune cells to the site of infection. This leads to the accumulation of immune cells in the lymph nodes, causing lymphadenopathy [15].

We hereby report two cases that presented with significant generalized peripheral lymphadenopathy. Both patients were Yoruba's and were hospitalized at the Federal Medical Center Owo, Ondo State, Nigeria.

### Case 1 (admitted 18 November 2022, discharged 8 December 2022)

#### Patient 1

Patient is a 27-year-old woman, secondary school leaver who works as a storekeeper, resides in Ondo State, was referred from St Louis hospital following a positive Lassa fever result.

##### Presenting complaints

- Fever × 1 week
- Passage of watery stool × 1 day

The fever was said to be high grade, continuous and present most times of the day. Fever was temporarily relieved by antipyretics. The patient also presented with watery stool, not mucoid or blood stained.

There was history of weakness, anorexia, headache, vomiting and contact with a co-worker with febrile illness. Patient 1 also said that there were rats in her home and workplace.

Prior to presentation she received anti-malaria and antibiotics.

History of hypertension, diabetes mellitus, peptic ulcer and asthma were not contributory.

###### Examination

Upon examination, patient 1 presented as acutely ill- looking, febrile, lethargic, not pale, anicteric, acyanosed and not dehydrated. The patient had cervical and axillary lymphadenopathy but no pedal edema.

Lymph node measures about 1 cm × 0.5 cm, not tender, rubbery, mobile, and not attached to underlying or overlying structures.

- Chest: RR 20 cpm (20–26)
- SPO_2_: 96% (92–97)
- Cardiovascular examination: PR - 69–114 bpm, BP 103/97–133/93 mmHg (range).

Patient 1, was given intranasal oxygen after 5 days on admission, when SPO_2_ dropped to 89–90%

###### Investigations results

- RBS 7.8 mmol/l (normal value)
- Anti-HCV, HBSag, HIV and VDRL were all normal.
- The blood culture result, which yielded growth of *staphylococcus aureus*, shows the presence of superimposed bacterial infection with sensitivity to ofloxacin, ceftriaxone, levofloxacin and gentamicin.
- The patient tested negative to COVID-19 PCR test.

A full blood count was taken (Table 1). These results clearly show the presence of lymphopenia, thrombocytopenia, monocytosis, mild neutrophilia and a normal haematocrit percentage using the normal reference values.

**Table 1.** The Full Blood Count results of patient 1.

	19 November 2022	24 November 2022	Normal reference value
WBC	5.09 × 10^3^/ul	6.74 × 10^3^	4–11 × 10^3^/ul
HCT	36.3%	33%	36–47%
PLT	60 × 10^3^/ul	203 × 10^3^	150–400 × 10^3^/ul
Lym	16.9%	11.3%	20–35%
Mon	51%	8.3%	3–8%
Neut	78.9%	76.9%	55–70%

HCT: Hematocrit; PLT: Platelet; WBC: White blood cell.

The electrolytes, blood, urea, nitrogen and creatinine were also investigated (Table 2). The results show hyponatremia at presentation. The later result showed a normal value post correction.

**Table 2.** Patient 1 electrolyte, blood, urea, nitrogen and creatinine results.

	19 November 2022	24 November 2022	Normal reference value
Na	128	136	135–145 mmol/l
K	3.8	3.4	3–4.5 mmol/l
BUN	3.2	2.8	2.1–8.5 mmol/l
CRE	54	58	62–106 umol/l

BUN: Blood, urea, nitrogen; CRE: Creatinine.

Patient 2 liver function tests revealed that AST was mildly elevated at presentation (Table 3). Alanine aspartate and alkaline phosphatase were missing results as they could not be analyzed due to absence of reagents.

**Table 3.** the liver Function Test of patient 1.

	19 November 2022	24 November 2022	Normal reference value
ALP	64	–	30–120 units/l
AST	174	–	0–35 U/l
ALB	30	20 g/l	35–50 g/l
TP		58 g/dl	64–83 g/dl

ALB: Albumin; ALP: Alkaline phosphatase; ALT: Alanine transaminase; AST: Aspartate aminotransferase.

Patient 1 was placed on the following medications: intravenous (iv) ribavirin, iv ceftriaxone, iv flagyl, iv promthazine, iv tranexemic acid. After 3 days on admission, fever persisted, tabs fluconazole, iv genticine, iv ciprofloxacin were prescribed. When fever persisted further, then ceftriaxone was discontinued with genticine while iv cefuroxime was commenced. Despite all the intervention, patient was still having temperature spikes until 3 days prior to discharge. She was also planned for LN biopsy, but was not done due to hypoalbuminaemia.

### Case 2 (admitted 19 November 2022, discharged 4 December 2022)

Patient 2 is a 43year old businessman (and animal farmer) who resides at Akure, presented following referral from a FMC Akure annex with

#### Presenting complaints

- Passage of watery stool × 1 week
- Generalized body weakness ×1 week
- Fever ×1 week
- Bilateral scrotal swelling ×2 months

Fever was said to be high grade, present throughout the day, with associated chills and rigor and was transiently relieved with antipyretics.

Patient 2 also had watery stools, about 3–4 episodes daily nonmucoid, nonblood stained. Patient at about the same time, complained of generalized body weakness which was gradual in onset and progressed till presentation. Patient 2 presented with background bilateral scrotal swelling, which has been ongoing in the last 2 years, there was no lower urinary tract symptoms. Patient 2 was reviewed by the urologist for the bilateral Hydrocele and was referred for a hydrocelectomy, after Lassa fever infection has been treated.

History of hypertension, diabetes mellitus, peptic ulcer and asthma were not contributory to the presenting complaints.

##### Examination

Patient 2 examination revealed a conscious, acutely ill-looking, febrile, pale, anicteric, acyanosed, mildly dehydrated male with cervical and axillary lymphadenopathy, discrete, nontender and measuring between 2×1 cm, nil pedal edema.

- Temperature: 36.3 degree Celsius (36.3–39.7)
- Chest: RR 24cpm (20–28), SPO2 96–100%
- Cardiovascular system: PR- 96 bpm (63–99), BP 120/80 mmhg (99/67–161/101 mmHg)
- Abdomen: full moves with respiration, nil areas of tenderness, liver and spleen not enlarged, kidneys not ballotable.

External genitalia: revealed cystic scrotal swelling, nontender, with differential warmth on palpation. The upper limit of the cystic scrotal swelling can be delineated and also trans-illuminates on application of light.

A digital rectal examination was deferred due to discomfort to the patient.

##### Investigations results

The initial values of the full blood count show mild anaemia, thrombocytopaenia, neutropenia and lymphocytosis (Table 4). The later values show an improvement in these parameters.

**Table 4.** Full blood count results for patient 2.

	22 November 2022	28 November 2022	Normal reference values
WBC	10.81 × 10^3^/ul	8.66	4–11 × 10^3^/ul
HGB	9.0 g/dl	9.0	11.5–16.5 g/dl
HCT	37.7%	29.0%	36–47%
Plt	145 × 10^3^/ul	214	150–400 × 10^3^/ul
Lym	43.1%	24.3%	20–35%
Mon	0.6%	7.7%	3–8%
Neut	49.0%	67.9%	55–70%
Baso	0.97%	0.0	0.0–0.1 × 10^3^/ul

HCT: Hematocrit; HGB: Hemoglobin; WBC: White blood cell.

Table 5 shows that the patient creatinine was elevated as at the time of presentation.

**Table 5.** Patient 2 electrolyte, blood, urea, nitrogen and creatinine results.

	22 November 2022	28 November 2022	Normal reference Value
Na	130	136	135–145 mmol/l
K	4.9	3.5	3–4.5 mmol/l
tCO2	23	18	23–30 mmol/l
Ca	3.4	2.3	2.12–2.4 mmol/l
Cl	103	110	102–109 mmol/l
BUN	6.4	5.6	2.1–8.5 mmol/l
CRE	162	123	62–106 umol/l

BUN: Blood, urea, nitrogen; CRE: Creatinine.

From the liver function tests, albumin and ALP were remarkably low and high respectively (Table 6).

**Table 6.** Liver function test of patient 2.

	22 November 2022	28 November 2022	Normal reference value
ALB	22	–	35–50 g/l
TP	–	–	64–83 g/dl
ALP	335	20	30–120 U/l
ALT	–	58	5–40 U/l
AST	–	160	0–35 U/l

ALB: Albumin; ALP: Alkaline phosphatase; ALT: Alanine transaminase; AST: Aspartate aminotransferase.

##### Serology

Patient 2 serology tests (HBsAg, HCV, VDRL and HIV) were all normal.

An injection abscess over the right gluteus was revealed with a mass measuring approximately 8×10cm. The mass was tender with differential warmth for which an ice pack was applied.

##### Medications given

Iv ribavirin, iv ceftriaxone, iv metronidazole, when fever persisted, iv ciprofloxacin, while iv metronidazole was discontinued after 1 week on admission. Other medications given were tabs fansidar, tabs amlodipine, iv paracaretamol, iv artesunate, iv albumin, caps astyfer.

Patient was also planned for lymph node biopsy but was not done due to hypoalbuminaemia and anaemia.

Patient 2 was transfused with a unit of pack cell prior to discharge due to anemia. He was subsequently discharged for follow-up.

## Discussion

Lymphadenopathy is a common finding in patients with viral infections, although not widely reported in Lassa fever. In Lassa fever, lymphadenopathy can be caused by the virus itself or as a result of the body's immune response to the infection. The pathogenesis of lymphadenopathy in Lassa fever is not fully understood, but it is believed to involve the activation of T-cells in the lymph nodes [14, 15].

The Lassa virus is known to infect immune cells such as dendritic cells, macrophages, and T-cells. When these cells become infected, they release cytokines and chemokines that attract other immune cells to the site of infection. This leads to the accumulation of immune cells in the lymph nodes, causing lymphadenopathy [15].

In addition to direct viral infection of immune cells, Lassa fever also triggers an overreaction of the T-cell section of the lymph nodes. T-cells play a critical role in the immune response to viral infections. However, in some cases, the immune response can become dysregulated, leading to excessive activation of T-cells and the release of large amounts of pro-inflammatory cytokines. This phenomenon is known as a cytokine storm and can cause tissue damage and organ failure [16].

While a cytokine storm can be harmful, it can also be beneficial in fighting viral infections. The release of pro-inflammatory cytokines attracts other immune cells to the site of infection, which helps to clear the virus. In the case of Lassa fever, lymphadenopathy may be a result of this beneficial immune response [15].

Currently, ribavirin is the standard treatment for Lassa fever, however there are debates as to its efficacy in the management of patients with Lassa fever [17]. We have reported two cases of Lassa fever in patients who presented with lymphadenopathy. Patients were successfully treated with Ribavirin.

## Limitations

Lymph node biopsy for histology and immunohistochemistry test could not be done for these patients as at the time it was requested, due to anaemia and hypoalbuminaemia, which are contraindications to the procedure, the center also do not have facility for immunohistochemistry.

## Conclusion

Lymphadenopathy is a common finding in viral infections but under-reported in Lassa fever patients. Lymphadenopathy is thought to be caused by the activation of T-cells in the lymph nodes. This activation can be a result of direct viral infection of immune cells or as a response to the virus. While excessive activation of T-cells can lead to harmful effects, it can also be beneficial in fighting the virus. Early diagnosis and commencement of Ribavirin is reported to reduce the case fatality rate [17].

This case reports have demonstrated evidence to support the presence of lymphadenopathy among Lassa fever patients and therefore, should be sort for and properly evaluated by physicians managing cases of Lassa fever.

**Summary points**

- The authors have presented two cases of Lassa fever.
- Both patients presented with lymphadenopathy that were successfully treated with ribavirin.
- This case report demonstrates evidence that lymphadenopathy in Lassa fever patients may be more common than previously assumed.
- The authors hope physicians will begin to look out for lymphadenopathy as a sign in Lassa fever patients.
